# Diagnostic accuracy of dermoscopy for onychomycosis: A systematic review

**DOI:** 10.3389/fmed.2022.1048913

**Published:** 2022-10-31

**Authors:** Sophie Soyeon Lim, Laura Hui, Jungyoon Ohn, Youngjoo Cho, Choon Chiat Oh, Je-Ho Mun

**Affiliations:** ^1^Alfred Health, Melbourne, VIC, Australia; ^2^Department of Dermatology, Singapore General Hospital, Singapore, Singapore; ^3^Department of Dermatology, Seoul National University College of Medicine, Seoul, South Korea; ^4^Institute of Human-Environment Interface Biology, Seoul National University, Seoul, South Korea; ^5^Department of Applied Statistics, Konkuk University, Seoul, South Korea

**Keywords:** fungal nail infection, onychomycosis, dermoscopy, fungal melanonychia, onychoscopy

## Abstract

**Background:**

Dermoscopy is a non-invasive adjuvant diagnostic tool that allows clinicians to visualize microscopic features of cutaneous disorders. Recent studies have demonstrated that dermoscopy can be used to diagnose onychomycosis. We performed this systematic review to identify the characteristic dermoscopic features of onychomycosis and understand their diagnostic utility.

**Methods:**

We searched the Medline, Embase, Scopus, and Cochrane databases from conception until May 2021. Studies on the dermoscopic features of onychomycosis were screened. The exclusion criteria were as follows: fewer than 5 cases of onychomycosis, review articles, and studies including onychomycosis cases that were not mycologically verified. Studies on fungal melanonychia were analyzed separately. We adhered to the MOOSE guidelines. Independent data extraction was performed. Data were pooled using a random effects model to account for study heterogeneity. The primary outcome was the diagnostic accuracy of the dermoscopic features of onychomycosis. This was determined by pooling the sensitivity and specificity values of the dermoscopic features identified during the systematic review using the DerSimonian-Laird method. Meta-DiSc version 1.4 and Review Manager 5.4.1 were used to calculate these values.

**Results:**

We analyzed 19 articles on 1693 cases of onychomycosis and 5 articles on 148 cases of fungal melanonychia. Commonly reported dermoscopic features of onychomycosis were spikes or spiked pattern (509, 30.1%), jagged or spiked edges or jagged edge with spikes (188, 11.1%), jagged proximal edge (175, 10.3%), subungual hyperkeratosis (131, 7.7%), ruins appearance, aspect or pattern (573, 33.8%), and longitudinal striae (929, 54.9%). Commonly reported features of fungal melanonychia included multicolor (101, 68.2%), non-longitudinal homogenous pigmentation (75, 50.7%) and longitudinal white or yellow streaks (52, 31.5%).

**Conclusion:**

This study highlights the commonly identified dermoscopic features of onychomycosis. Recognizing such characteristic dermoscopic features of onychomycosis can assist clinicians diagnose onychomycosis by the bedside.

## Introduction

Dermoscopy is a non-invasive diagnostic tool that helps clinicians to visualize microscopic features of cutaneous disorders, including skin cancers, connective tissue disorders and inflammatory dermatologic conditions, that are not discernible on naked eye examination (1–3). Consequently, it optimizes diagnostic accuracy and minimizes the need for unnecessary biopsies (4).

Onychomycosis is a communicable fungal nail infection caused by dermatophytes, non-dermatophyte molds, and yeasts. It is the most common nail disorder worldwide and severe disease can cause significant nail dystrophy and pain (5). Fungal melanonychia is a rare manifestation of a fungal nail infection, which presents with brown-black pigmentation of the nail unit. Accurate diagnosis of fungal nail disorders is important as systemic treatments are required for at least 2–3 months and topical treatments for more than 12 months. Misdiagnosis should be avoided, as systemic treatments risk hepatic damage (6) and unnecessary economic burden on the healthcare system. Clinically, onychomycosis may resemble traumatic onycholysis, nail psoriasis, or trachyonychia, and differentiating fungal melanonychia from nail melanoma is crucial. Dermoscopy can help to identify onychomycosis and fungal melanonychia at the bedside. Therefore, we conducted a systematic review to identify the characteristic dermoscopic features of onychomycosis and melanonychia, as well as a meta-analysis to determine the diagnostic performance and accuracy of dermoscopy in diagnosing onychomycosis.

## Methods

This study adhered to the Meta-analyses of Observational Studies in Epidemiology (MOOSE) statement, with appropriate adjustments made as per the recommendations for systematic reviews and meta-analyses of diagnostic test accuracy (7, 8). The study protocol is registered in PROSPERO (Reg. No.: CRD42021268430).

### Literature search

Ovid MEDLINE (including Epub Ahead of Print, In-Process, and Other Non-Indexed Citations), Embase, Scopus, and the Cochrane Central Register of Controlled Trials were searched from inception to May 2021 by three reviewers (SSL, JO, LHLY). The research question was in patients with onychomycosis (P), what are the common dermoscopic features (I) that add to clinical examination (C) in diagnosing onychomycosis (O). Therefore, search terms included “dermoscopy” or synonyms (including dermatoscopy, videodermoscopy, onychoscopy and epiluminescence microscopy) and “onychomycosis” or synonyms (including tinea unguium). Medical subject headings (MeSH) terms were also included.

### Eligibility criteria

All published studies involving at least five cases of mycologically proven onychomycosis with dermoscopic findings were included. Studies reporting fewer than five cases were excluded due to risk of selection bias. Studies of fungal melanonychia were analyzed separately.

### Study selection and data extraction

Three reviewers (SSL, JO, and LHLY) independently screened the titles and abstracts of all identified articles, and then screened the full text of potentially eligible articles. Non-English articles were screened by reviewing their titles and abstracts translated in English. Duplicate studies and review articles were excluded. None of the cases required a fourth author (JHM) to resolve any disagreement. The parameters extracted from each article included the first author's surname, date of publication, journal name, number of onychomycosis or fungal melanonychia cases, number of control cases, type of control cases (e.g., healthy or psoriasis), definition and prevalence of dermoscopic features, as well as their sensitivity and specificity if reported. Study authors were not contacted.

### Risk of bias assessment

Two reviewers (SSL and JO) appraised the articles according to the Quality Assessment of Diagnostic Accuracy Studies (QUADAS2) guidelines (9).

### Statistical analysis of the primary study outcome

The primary study outcome was diagnostic accuracy of the common dermoscopic features of onychomycosis. This was measured by pooling the sensitivity and specificity values using the DerSimonian-Laird method. We used a random-effects model to account for study heterogeneity. Pooled sensitivity and specificity values and their 95% confidence intervals (CI), forest plots, and summary receiver operating characteristics (SROC) curves were generated using Meta-DiSc version 1.4 (Hospital Ramon y Cajal and Universidad Complutense de Madrid) and Review Manager 5.4.1 (Cochrane, Oxford, UK).

## Results

### Literature search and included studies

**Figure 1:**
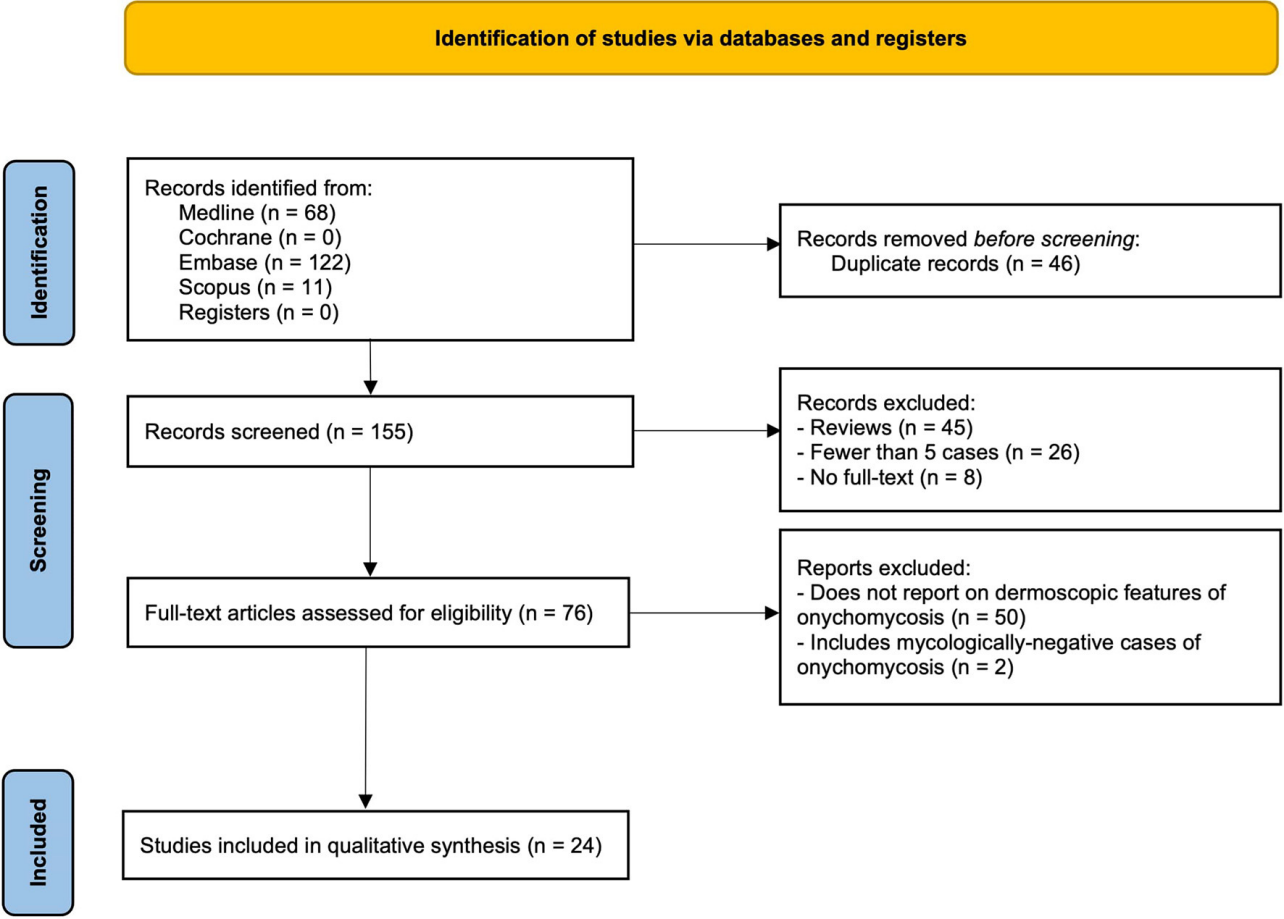
Flow diagram illustrating search strategy.

A total of 201 articles were identified, of which 46 were duplicates (Figure 1). Of the 155 screened articles, 24 were full-text articles discussing common dermoscopic features in five or more cases of mycologically proven fungal nail disease. The characteristics of the 24 eligible studies are summarized in Table 1. Nineteen articles were on onychomycosis and five were on fungal melanonychia. Of the 19 onychomycosis articles, 11 had a control group consisting of nail psoriasis, traumatic onycholysis, and healthy or mycologically negative nails. A meta-analysis was performed on the data with controls. However, it was not conducted for fungal melanonychia, as only two of the five fungal melanonychia articles had a control group.

**Table 1:** Characteristics of the eligible studies.

**First author and journal**	**Publication date**	**Study population**	**Number of patients**	Number of controls (Y/N)^*^	**Control characteristics**
Abdallah, *J Cosmet Dermatol*	2020	Onychomycosis	40	N	-
Ankad, *Indian Dermatol Online J*	2020	Onychomycosis	20	Y, 40	Nail psoriasis (*n =* 35), traumatic onycholysis (*n =* 5)
Bhat, *Dermatol Pract Concept*	2018	Onychomycosis	81	N	-
Bodman, *J Am Podiatr Med Assoc*	2017	Onychomycosis	35	Y, 17	Mycologically negative nails
Chetana, *Int J Dermatol*	2018	Onychomycosis	234	N	-
De Crignis, *Int J Dermatol*	2014	Onychomycosis	336	N	-
El-Hoshy, *Eur J Dermatol*	2015	Onychomycosis	40	Y, 40	Healthy nails
Elfar, *J Egypt Women Dermatol*	2015	Onychomycosis	17	Y, 15	Traumatic onycholysis (*n =* 9), dermatophyte-negative psoriasis (*n =* 6)
Elmas, *Postepy Dermatol Alergol*	2020	Fungal melanonychia	42	N	-
Islamoglu, *Erciyes Med J*	2019	Onychomycosis	100	N	-
Jesus-Silva, *Dermatol Pract Concept*	2015	Onychomycosis	155	N	-
Jo, *Br J Dermatol*	2018	Onychomycosis	30	Y, 30	Trachyonychia
Kayarkatte, *Indian J Dermatol Venereol Leprol*	2020	Onychomycosis	88	Y, 12	Mycologically negative nails
Kaynak, *Arch Dermatol*	2018	Onychomycosis	149	Y, 56	Mycologically negative nails
Kilinc Karaarslan, *Clin Exp Dermatol*	2015	Fungal melanonychia	20	N	-
Kim, *Ann Dermatol*	2020	Fungal melanonychia	20	14	Subungual melanoma
Maatouk, *Curr Med Mycol*	2019	Onychomycosis	45	N	-
Nada, *Arch Dermatol*	2020	Onychomycosis	80	Y, 40	Healthy nails
Nargis, *Indian Dermatol Online J*	2018	Onychomycosis	60	N	-
Ohn, *J Am Acad Dermatol*	2016	Fungal melanonychia	18	Y, 62	Nail matrix naevus (*n =* 27), melanoma (*n =* 11), melanocytic activation (*n =* 24)
Piraccini, *J Eur Acad Dermatol Venereol*	2013	Onychomycosis	37	Y, 13	Traumatic onycholysis
Ramos Pinheiro, *J Eur Acad Dermatol*	2020	Onychomycosis	110	Y, 82	Traumatic onycholysis
Starace, *Mycoses*	2021	Fungal melanonychia	48	N	-
Yadav, *Indian J Dermatol*	2016	Onychomycosis	36	Y, 10	Nail psoriasis

* Y: Yes, N: No.

### Dermoscopic features of onychomycosis

Nineteen studies reported dermoscopic features of 1,693 cases of onychomycosis. Commonly identified dermoscopic features of onychomycosis were spikes or spiked pattern (481, 28.4%) (10–18), jagged or spiked edges or jagged edge with spikes (188, 11.1%) (19–25), jagged proximal edge (175, 10.3%) (10, 12, 16, 18), subungual hyperkeratosis (131, 7.7%) (15, 19, 20, 25, 26), ruins appearance, aspect or pattern (573, 33.8%) (15, 19, 22, 24, 27, 28), and longitudinal striae (929, 54.9%) (10–18, 20–23, 27) (Table 2). Other dermoscopic findings included distal irregular termination (331, 19.6%) (10–12, 14–16, 18, 20, 22) and aurora borealis pattern (293, 17.3%) (11, 12, 15, 17, 20, 23). Frequently described color changes were homogenous leukonychia (304, 18.0%) (12, 15, 16, 20, 22, 23, 28), yellow (216, 12.8%) (13, 15, 16, 23, 26) and brown (212, 12.5%) (12, 15, 16, 22, 23, 26).

**Table 2:** Dermoscopic features of onychomycosis reported in one or more articles.

**First author and publication date**	**Sample size**	**Jagged edge with spikes**	**Spiked pattern**	**Jagged proximal edge**	**Distal streaks**	**Longitudinal striae**	**Subungual hyperkeratosis**	**Ruin appearance/ aspect/pattern **	**Black dots and globules**	**Distal irregulartermination**	**Dryness & scaling of adjacent skin **	**Linear edge**	**Onycholysis**	**Pits**	**Splinter hemorrhages**	**White/ homogenous leukonychia **	**Punctate leukonychia**	**Black**	**Brown**	**Orange**	**Yellow**	**Chromonychia**	**Aurora borealis**
Abdallah et al. (10)	40		24 (60%)	20 (50%)		33 (82.5%)				19 (47.5%)		0 (0%)										36 (90%)	
Ankad et al. (19)	20	18 (90%)					5 (25%)	13 (65%)						0 (0%)	1 (5%)								
Bhat et al. (11)	81		69 (85.19%)			63 (77.78%)				33 (40.74%)													63 (77.78%)
Bodman (20)	35	34 (97.1%)				31 (88.6%)	22 (62.9%)		23 (65.7%)	27 (77.1%)	13 (37.1%)	2 (5.7%)	24 (68.6%)			28 (80.0%)						32 (86.4%)	27 (71.1%)
Chetana et al. (12)	234		101 (43.16%)	70 (29.91%)		115 (49.15%)			Black dots: 44 (18.80%) Black globules 35 (15.38%)	81 (34.62%)		8 (3.42%)		13 (5.56%)	6 (2.56%)	98 (41.88%)		79 (33.76%)	127 (54.27%)	46 (19.66%)	104 (44.44%)		61 (26.07%)
De Crignis et al. (27)	336					267 (79.46%)		296 (88.09%)															
El-Hoshy et al. (13)	40		40 (100%)			33 (82.5%)																	
Elfar et al. (21)	17	13 (76.47%)				16 (94.12%)						0 (0%)			2 (11.76%)								
Islamoglu et al. (22)	100	6 (6%)				66 (66%)		54 (54%)		20 (20%)					8 (8%)	28 (28%)	16 (16%)	10 (10%)	20 (20%)				
Jesus-Silva et al. (14)	155		39 (25.16%)			94 (60.65%)				67 (43.22%)		34 (21.94%)											
Jo et al. (26)	30				28 (93.3%)		22 (73.3%)						20 (66.7%)	2 (6.7%)					7 (23.3%)		28 (93.3%)		
Kayarkatte et al. (15)	88		76 (86.4%)			22 (25%)	75 (85.2%)	52 (59.1%)		72 (81.8%)	73 (83%)		85 (96.6%)			30 (34.1%)		8 (9.1%)	24 (27.3%)		44 (50%)	75 (85.2%)	17 (85%)
Kaynak et al. (28)	149							143 (95.97%)								88 (59.06%)	113 (75.84%)	33 (22.15%)					
Maatouk et al. (16)	45		25 (55.5%)	25 (55.5%)		31 (68.75%)				5 (11.1%)		2 (4.4%)		4 (8.8%)	2 (4.4%)	10 (22.2%)		0 (0%)	15 (33.3%)	6 (13.3%)	14 (31.1%)		
Nada et al. (17)	80		60 (75%)			66 (82.5%)																	76 (95%)
Nargis et al. (18)	60		47 (78.3%)	60 (100%)		60 (100%)				7 (11.7%)												23 (38.3%)	
Piraccini et al. (23)	37	37 (100%)				32 (86.49%)			23 (62.16%)			0 (0%)				22 (59.46%)		9 (24.32%)	19 (51.35%)	9 (24.32%)	26 (70.27%)		32 (86.49%)
Ramos Pinheiro et al. (24)	110	59 (53.6%)						15 (13.6%)					5 (4.55%)										
Yadav et al. (25)	36	21 (58.33%)					7 (19.44%)															13 (36.11%)	

**Figure 2:**
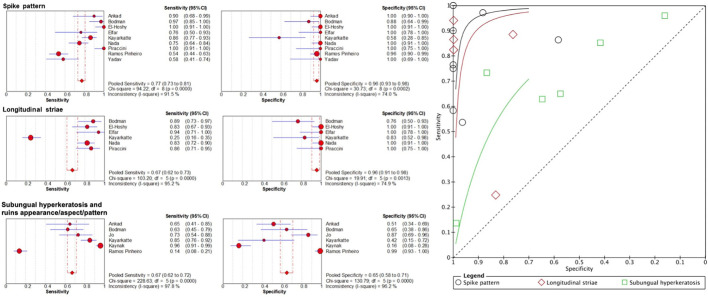
Sensitivity and specificity of important dermoscopic features of onychomycosis and their summary receiver operating characteristics (SROC) curves.

Terms with similar definitions or those used interchangeably were grouped for meta-analysis upon careful examination of the authors' definitions. When we grouped spikes or spiked pattern, jagged or spiked edges, distal streaks, jagged edge with spikes and jagged proximal edge as “spike pattern”, the pooled sensitivity was 77.3% (95% CI, 73.2–81.1%) and specificity was 96.2% (95% CI, 93.1–98.2%) (Figure 2). Pooled sensitivity of subungual hyperkeratosis and ruins appearance, aspect or pattern was 67.1% (95% CI, 62.5–71.5%) and specificity was 64.7% (95% CI, 58.1–70.8%). For longitudinal striae, pooled sensitivity was 67.3% (95% CI, 61.7–72.6%) and specificity 95.6% (95% CI, 90.7–98.4%).

### Dermoscopic features of fungal melanonychia

Five studies reported dermoscopic features of 148 cases of fungal melanonychia (Table 3) (29–31, 33). These cases demonstrated longitudinal white or yellow streaks (52, 35.1%) (31–33), nail surface scales (39, 33.1%) (31–33) and subungual hyperkeratosis (41, 27.7%) (31–33) (Table 3), which are also common dermoscopic features of onychomycosis. Homogenous pigmentation (75, 50.7%) (29, 30, 33) or longitudinal pigmentation (54, 36.5%) (29, 31–33) was frequently observed, and the most common colors were multicolor (101, 68.2%) (29–33), brown (84, 56.8%) (29, 31–33) and black (46, 31.1%) (29, 31–33). The pigmentation in melanonychia arising from a fungal infection tends to appear brown due to the production of fungal melanin *via* the pentaketide pathway (31). This is in contrast to melanomas, where melanin is made from tyrosine, and commonly appears as darkly pigmented and black. Findings that appear specific to fungal melanonychia, such as “reverse triangle” (30, 20.3%) (30–33), due to fungal invasion from the distal nail plate and “superficial transverse striation” (41, 27.7%) (29, 30, 33), were also reported. All cases had negative findings for melanoma, such as the lack of the Hutchinson sign (0%) (30–33) and triangular sign (0%) (31, 32).

**Table 3:** Dermoscopic features of fungal melanonychia.

		**Pattern**					**Pigmentation**		**Color**						**Melanonychia**				**Nail melanoma-associated patterns**		
**First author and publication date**	**Sample size**	**Subungual hyperkeratosis**	**White or yellow streaks**	**Nail surface scales**	**Reverse triangular pattern**	**Superficial transverse striation**	**Longitudinal**	**Homogenous**	**Multicolor (> 2)**	**Yellow**	**Black**	**Brown**	**Gray**	**Striata or Longitudinal**	**Distal partial diffuse**	**Prox partial diffuse**	**Distal linear**	**Total diffuse**	**Hutchinson's sign**	**Pseudo- Hutchinson's sign**	**Triangular sign**
Elmas et al. (29)	42					11 (26.1%)	4 (9.5%)	33 (78.5%)	38 (90.4%)		19 (45.2%)	21 (50%)	11 (26.1%)							4 (9.5%)	
Kilinc Karaaslan et al. (30)	20				2 (10%)	7 (35%)		20 (100%)	19 (95%)		1 (5%)			7 (35%)	5 (25%)	4 (20%)	2 (10%)	2 (10%)	0 (0%)	2 (10%)	
Kim et al. (31)	20	7 (35%)	18 (90%)	14 (70%)	10 (50%)		6 (30%)		13 (65%)	8 (40%)	10 (50%)	18 (90%)	0		7 (35%)	1 (5%)	4 (20%)	2 (10%)	0 (0%)	0 (0%)	0 (0%)
Ohn et al. (32)	18	10 (55.6%)	16 (88.9%)	13 (72.2%)	7 (38.9%)		8 (44.4%)	10 (55.6%)	16 (88.9%)	14 (77.8%)	10 (55.6%)	11 (61.1%)	3 (16.7%)						0 (0%)	1 (5.6%)	0 (0%)
Starace et al. (33)	48	24 (50%)	18 (37.5%)	22 (45.8%)	11 (22.9%)	23 (47.9%)	36 (75%)	12 (25%)	15 (31.3%)	5 (10.4%)	6 (12.5%)	34 (70.8%)	11 (22.9%)	20 (41.67%)	8 (16.7%)	2 (4.17%)	1 (2.08%)	17 (35.4%)	0 (0%)	0 (0%)	

### Quality assessment

The risk of bias in the eligible articles was evaluated according to the QUADAS2 guidelines (Table 4). Studies with “unclear” patient selection bias did not specify their method of patient selection, such as whether patients were recruited prospectively or retrospectively or whether patients were enrolled consecutively or randomly. Studies had a low risk of bias in terms of the index test (dermoscopy), reference standard (clear diagnosis of non-onychomycosis nails), flow, and timing. However, the risk of bias in the reference standard for one article was deemed high, as two cases with a positive potassium hydroxide result were not classified as onychomycosis as they primarily displayed features of other nail disorders (19). Studies had low applicability concerns with patient selection and reference standards, but two studies had high applicability concerns with the index test because they did not provide clear definitions or representative images for dermoscopic features (25, 27).

**Table 4:** Quality assessment of the included studies.

	**Risk of bias**				**Applicability**		
**First author and publication date**	**Patient selection**	**Index test**	**Reference standard**	**Flow and timing**	**Patient selection**	**Index test**	**Reference standard**
Abdallah et al. (10)	Unclear	Low	Low	Low	Low	Low	Low
Ankad et al. (19)	Low	Low	High	Low	Low	Low	Low
Bhat et al. (11)	Low	Low	N/A (no control group)	Low	Low	Low	Low
Bodman (20)	Unclear	Low	Low	Low	Low	Low	Low
Chetana et al. (12)	Low	Low	N/A (no control group)	Low	Low	Low	Low
De Crignis et al. (27)	Low	Low	N/A (no control group)	Low	Low	High	Low
El-Hoshy et al. (13)	Unclear	Low	Low	Low	Low	Low	Low
Elfar et al. (21)	Low	Low	Low	Low	Low	Low	Low
Elmas et al. (29)	Low	Low	N/A (no control group)	Low	Low	Low	Low
Islamoglu et al. (22)	Low	Low	N/A (no control group)	Low	Low	Low	Low
Jesus-Silva et al. (14)	Unclear	Low	Low	Low	Low	Low	Low
Jo et al. (26)	Low	Low	Low	Low	Low	Low	Low
Kayarkatte et al. (15)	Unclear	Low	Low	Low	Low	Low	Low
Kaynak et al. (28)	Unclear	Low	Low	Low	Low	Low	Low
Kilinc Karaaslan et al. (30)	Unclear	Low	N/A (no control group)	Low	Low	Low	Low
Kim et al. (31)	Unclear	Low	Low	Low	Low	Low	Low
Maatouk et al. (16)	Low	Low	N/A (no control group)	Low	Low	Low	Low
Nada et al. (17)	Low	Low	Low	Low	Low	Low	Low
Nargis et al. (18)	Low	Low	N/A (no control group)	Low	Low	Low	Low
Ohn et al. (32)	Unclear	Low	Low	Low	Low	Low	Low
Piraccini et al. (23)	Low	Low	Low	Low	Low	Low	Low
Ramos Pinheiro et al. (24)	Low	Low	Low	Low	Low	Low	Low
Starace et al. (33)	Low	Low	N/A (no control group)	Low	Low	Low	Low
Yadav et al. (25)	Unclear	Low	Low	Low	Low	High	Low

## Discussion

The role of dermoscopy is well established in diagnosing cutaneous malignancies such as malignant melanoma and non-melanoma skin cancers (34, 35). Its use expands to various inflammatory and infectious disorders, including onychomycosis. By conducting a systematic review of 19 articles on 1,693 cases of onychomycosis and 5 articles on 148 cases of fungal melanonychia, we could enlarge the sample size and thus the statistical power to identify the dermoscopic features with diagnostic utility. Recognizing common dermoscopic features of onychomycosis can help clinicians to expedite accurate diagnosis and management. The most frequently reported patterns in onychomycosis included spikes or spiked patterns, ruins appearance, aspect or pattern and longitudinal striae. After pooling the dermoscopic terminology that were closely related or used interchangeably, “spike pattern” and longitudinal striae had high specificity (96.2 and 95.6%, respectively) and moderate sensitivity (77.3 and 67.3%, respectively) for onychomycosis. Detecting these features can raise clinicians' suspicion of onychomycosis and expedite further investigations. Ruins appearance, aspect or pattern and subungual hyperkeratosis had moderate sensitivity (71.6%) and specificity (64.7%) for onychomycosis as these features can also be observed in other nail disorders including nail psoriasis and allergic contact dermatitis. Other dermoscopic features characterizing onychomycosis were distal irregular termination, aurora borealis, homogenous leukonychia, and brown discoloration.

We also found that the most frequently described dermoscopic features of fungal melanonychia were longitudinal white or yellow streaks and nail surface scales. Unlike melanocytic melanonychia, fungal melanonychia is characterized by non-longitudinal homogenous pigmentation and reverse triangular patterns (32). Moreover, our data demonstrate that subungual hyperkeratosis frequently occurs in fungal melanonychia.

This study has some limitations. There was considerable heterogeneity in the study design and terminology definitions of the enrolled studies, which may have limited the strength of our study. We sought to clarify dermoscopic terminology by identifying commonly used terms and narrowing their definitions to accurately pool and compare the findings. Future studies with standardized terminology are necessary, ideally through an expert panel, to facilitate clear communication among clinicians.

To our knowledge, this is the first systematic review of dermoscopic features of onychomycosis. Given the limited sample sizes of existing studies on this topic, pooling their results provides us an overview of the most common features of onychomycosis and the frequency at which they present in patients. Understanding these characteristic dermoscopic features of onychomycosis can assist clinicians diagnose onychomycosis by the bedside.

## Data availability statement

The original contributions presented in the study are included in the article/supplementary material, further inquiries can be directed to the corresponding author.

## Author contributions

J-HM and CO: conceptualization, resources, and supervision. J-HM: methodology and project administration. YC, J-HM, and CO: validation. SL, JO, and LH: formal analysis. SL and LH: investigation, data curation, writing—original draft, and visualization. JO, YC, CO, and J-HM: writing—review and editing. All authors: software. All authors contributed to the article and approved the submitted version.

## Conflict of interest

The authors declare that the research was conducted in the absence of any commercial or financial relationships that could be construed as a potential conflict of interest.

## Publisher's note

All claims expressed in this article are solely those of the authors and do not necessarily represent those of their affiliated organizations, or those of the publisher, the editors and the reviewers. Any product that may be evaluated in this article, or claim that may be made by its manufacturer, is not guaranteed or endorsed by the publisher.
